# Nutritional status and prognosis in metastatic colorectal cancer: a cohort study

**DOI:** 10.1590/1806-9282.20250238

**Published:** 2025-07-07

**Authors:** Tanju Kapagan, Nilufer Bulut, Beyza Arslansoy, Zozan Ozcalimli, Mehmet Turkmencalikoglu, Ece Yontan, Cagla Ecem Kılıc, Beyza Canan Ozkan Kardes, Gokmen Umut Erdem

**Affiliations:** 1 Başakşehir Çam and Sakura City Hospital, Department of Internal Medicine, Division of Medical Oncology – İstanbul, Türkiye.; 2 Başakşehir Çam and Sakura City Hospital, Department of Internal Medicine – İstanbul, Türkiye.

**Keywords:** Nutrition assessment, Nutritional status, Colorectal neoplasms, Survival

## Abstract

**OBJECTIVE:**

Colorectal cancer is a common and often fatal malignancy, and predicting survival can help guide treatment decisions. This study aimed to investigate the impact of clinicopathological characteristics and nutritional status on survival in patients with metastatic colorectal cancer receiving first-line treatment with 5-fluorouracil in combination with monoclonal antibodies (bevacizumab, cetuximab, or panitumumab).

**METHODS:**

This prospective, non-interventional, observational, single-center study included 150 adult patients with metastatic colorectal cancer undergoing first-line therapy. Demographic data, cancer-related characteristics, laboratory parameters, and nutritional status assessed via the Mini Nutritional Assessment–Short Form were recorded prior to treatment initiation. Mini Nutritional Assessment–Short Form scores of 12–14, 8–11, and 0–7 were used to define normal nutritional status, risk of malnutrition, and malnutrition, respectively.

**RESULTS:**

The mean ages of patients without and with malnutrition were 60±11 and 62±11 years, respectively. Median progression-free survival and overall survival were 12 and 22 months, respectively. Multivariate analysis identified alcohol history (HR=2.83; 95%CI 1.27–6.34; p=0.011) and Mini Nutritional Assessment–Short Form≤7 (HR=1.93; 95%CI 1.09–3.45; p=0.025) as significant predictors of shorter progression-free survival. Mini Nutritional Assessment–Short Form ≤7 (HR=2.96; 95%CI 1.80–4.89; p<0.001) was also significantly associated with decreased overall survival.

**CONCLUSION:**

Among patients with metastatic colorectal cancer receiving first-line therapy, malnutrition (Mini Nutritional Assessment–Short Form≤7) and a history of alcohol use were associated with poorer survival outcomes. These findings underscore the importance of routine nutritional assessment and support the need for further prospective multicenter studies to validate these results.

## INTRODUCTION

Colorectal cancer (CRC) is a major cause of morbidity and mortality worldwide, and is the third most common cancer in both men and women^1^. According to the Surveillance, Epidemiology, and End Results (SEER) program, an estimated 152,810 new cases of CRC will be diagnosed in the US in 2024, with 53,010 deaths due to the disease^2^. Approximately 22% of the patients with CRC are diagnosed during the metastatic stage^3^. Patients diagnosed with metastatic CRC (mCRC) have a significantly worse prognosis compared to those with localized disease^4^.

In patients diagnosed at the metastatic stage, the poor prognosis poses significant challenges in disease management. This also negatively affects treatment response rates and patient survival outcomes^5^. In this context, the impact of patients’ baseline nutritional status on disease prognosis is becoming increasingly crucial.

Nutritional status has a significant impact on the survival rate and quality of life of cancer patients. Studies have shown that malnourished patients have a reduced ability to respond to cancer treatments, an increased risk of infections, and prolonged hospital stays^6,7^. In cancer types such as CRC, which are associated with metabolic dysfunction, investigating how patients’ baseline nutritional status affects survival, particularly in the context of metastatic disease, may contribute to the customization of treatment strategies.

This study investigated the correlation between nutritional status, progression-free survival (PFS), and overall survival (OS) in patients with mCRC receiving 5-fluorouracil (5-FU) in combination with monoclonal antibodies (bevacizumab, cetuximab, and panitumumab) as first-line treatment.

## METHODS

### Informed consent

This study was performed in line with the principles of the Declaration of Helsinki. Approval was granted by the Ethics Committee of Basaksehir Cam and Sakura City Hospital (KAEK/2022.03.93). Written informed consent was obtained from all patients before the conduct of the study.

### Study population

This prospective, non-interventional, observational, single-center study enrolled patients with de novo or recurrent mCRC who were admitted to the medical oncology outpatient clinic between 2022 and 2025. All patients received 5-FU-based chemotherapy as first-line treatment. Patients were followed up until disease progression or death while on 5-FU-based treatment. Demographic data at the time of diagnosis, clinical characteristics (cancer subtypes, surgery history, palliative radiotherapy, and presence and location of metastasis), and nutritional statuses were recorded just before starting 5-FU-based therapy.

### Nutritional status evaluation

The Mini Nutritional Assessment-Short Form (MNA-SF) was used to assess patients’ malnutrition status. The MNA-SF consists of six screening criteria:

1. Food intake (0=severe decrease in food intake, 1=moderate decrease in food intake, 2=no decrease in food intake);
2. Unintentional weight loss (0=weight loss greater than 3 kg [6.6 lbs], 1=does not know, 2=weight loss between 1 and 3 kg [2.2 and 6.6 lbs], 3=no weight loss);
3. Mobility (0=bed or chairbound, 1=able to get out of bed/chair but does not go out, 2=goes out);
4. Psychological stress or acute illness (0=yes, 2=no);
5. Neuropsychological problems (0=severe dementia or depression, 1=mild dementia, 2=no psychological problems);
6. Body mass index (BMI) (0=BMI: less than 19, 1=BMI: 19 to less than 21, 2=BMI: 21 to less than 23, 3=BMI: 23 or greater).

MNA-SF scores of 12–14, 8–11, and 0–7 indicate normal nutritional status, malnutrition risk, and malnutrition, respectively.

The selection method and inclusion criteria for the patients were:

- Having de novo or recurrent metastatic disease;
- >18 years of age;
- Agreeing to receive 5-FU-based therapy as first-line treatment.

On the other hand, the exclusion criteria of the study were:

- Diagnosis of a second primary cancer;
- Presence of high-grade hematologic abnormalities (hemoglobin<8 g/dL), thrombocytopenia (platelet count<75,000×10^9^/L), or neutropenia (neutrophil count<1,000×10^9^ /L);
- Eastern Cooperative Oncology Group Performance Status (ECOG PS) of 3–4.

In total, 150 CRC patients, 91 males and 59 females, were included in the study.

### Statistical analysis

Statistical analyses were performed using SPSS Statistics 27 (ver. 20.2.1.15749). For descriptive statistics, numbers and percentages were used for representing categorical variables. Mean, standard deviation, and minimum and maximum values were used for representing numerical variables. The proportions in independent groups were analyzed using the chi-square test. Comparisons of numerical variables in two separate groups were performed by the Mann-Whitney U test since the condition of normal distribution was not met. Cox proportional hazards models were used in univariate and multivariate analyses of factors affecting survival. For multivariate analysis, the "Enter" method was used. The hazard ratio (HR) was reported with the corresponding 95% confidence intervals (95%CIs). The endpoint for PFS was defined as clinical or radiological disease progression after starting nivolumab, and the endpoint for OS was defined as death after starting nivolumab or the date of last follow-up. Survival was analyzed using the Kaplan-Meier method, and the log-rank test was used for group comparison. Statistical significance was considered at p<0.05.

## RESULTS

### Clinicopathological and laboratory parameters

In our study, there were 87 non-malnourished and 63 malnourished patients. The median MNA-SF score (10 points vs. 6 points) was significantly higher in the non-malnourished group. Table 1 presents comprehensive information on the clinicopathological and laboratory parameters.

**Table 1:** Clinicopathological and laboratory parameters of the patients.

Variables		Non-malnourished group (MNA-SF >7)		Malnourished group (MNA-SF ≤7)		p-value
		n=87		n=63		
		n	%	n	%	
Age (years)*		60±11	28–81	62±11	29–82	0.839
Gender	Female	31	36	28	44	0.277
	Male	56	64	35	56	
ECOG PS	≤1	39	45	31	49	0.597
	>1	48	55	32	51	
Smoking history	Yes	29	48	15	47	0.951
	No	32	52	17	53	
Alcohol history	Yes	4	8	8	23	0.059
	No	48	92	27	77	
Chronic disease	Yes	37	43	32	51	0.316
	No	50	57	31	49	
KRAS	Positive	42	50	26	46	0.609
	Negative	42	50	31	54	
BRAF	Positive	3	4	0	0	0.275
	Negative	81	96	56	100	
MSI-H	Yes	4	5	1	2	0.395
	No	80	95	61	98	
Chemotherapy dose reduction	Yes	19	22	22	35	0.066
	No	68	78	40	65	
Blood group (Rh)	Rh+	75	89	45	80	0.139
	Rh-	9	11	11	20	
Blood group (ABO)	O	32	38	21	38	0.943
	A	34	41	21	38	0.494
	B	11	13	8	14	0.841
	AB	7	8	6	10	0.860
Tumor site	Rectum/rectosigmoid	43	49	26	41	0.323
	Colon	44	51	37	59	
Metastasis site	Liver	66	54	50	51	0.613
	Lung	25	21	20	21	0.691
	Bone	4	3	7	7	0.203
	Peritoneum	12	10	12	12	0.386
	Others	15	12	9	9	0.626
Measured/laboratory parameters		Median	Min–Max	Median	Min–Max	p-value
MNA-SF		6	3–7	10	8–14	0.001
Weight (kg)		74	45–110	65	30–94	0.568
Height (cm)		169	146–193	167	154–185	0.162
BMI (kg/m^2^)		26	17–40	24	13–39	0.429
CRP (mg/dL)		18	0–299	11	1–313	0.469
Albumin (g/dL)		41	21–51	40	20–50	0.504
Creatinine (mg/dL)		0.8	0.4–3.0	0.8	0.3–1.9	0.640
AST (IU/L)		18	5–106	19	8–113	0.163
ALT (IU/L)		15	2–136	15	4–91	0.979
Lactate dehydrogenase (U/L)		205	124–3,391	231	65–3,092	0.174
Hemoglobin (g/dL)		12	6–17	12	7–16	0.160
Platelet (109/L)		323	65–836	309	108–971	0.499
Leukocyte (109/L)		8.4	3.1–21.0	8.2	3.2–16.2	0.782

* Mean±SD; Min–Max; ALT: alanine aminotransferase; AST: aspartic transaminase; BMI: body mass index; BRAF: B-rapidly accelerated fibrosarcoma; CRP: C-reactive protein; ECOG PS: Eastern Cooperative Oncology Group Performance Status; KRAS: Kirsten rat sarcoma viral oncogene; Max: maximum; Min: minimum; MNA-SF: Mini Nutritional Assessment-Short Form; SD: standard deviation. Statistically significant values are denoted in bold

### Risk factors for progression-free survival

The median PFS of the patients was 12 months. In the univariate analysis, age, gender, BMI, ECOG PS, smoking history, chronic disease, liver metastasis, lung metastasis, bone metastasis, peritoneal metastasis, and RH blood group were not significantly associated with PFS. In the multivariate analysis, alcohol history (HR=2.83, 95%CI 1.27–6.34, p=0.011) and MNA-SF ≤7 points (HR=1.93, 95%CI 1.09–3.45, p=0.025) were identified as negative risk factors for PFS. Table 2 shows the factors affecting PFS.

**Table 2:** Univariate and multivariate analyses of characteristic parameters related to progression-free survival.

PFS		Univariate analysis		Multivariate analysis	
Characteristics	Category	HR (95%CI)	p	HR (95%CI)	p
Age	<65 vs. ≥65	1.28 (0.88–1.87)	0.198		
Gender	Female vs. male	0.99 (0.69–1.47)	0.987		
BMI	≤25.3 vs. >25.3	0.82 (0.56–1.19)	0.287		
ECOG PS	≤1 vs. >1	0.99 (0.68–1.45)	0.986		
Smoking history	Yes vs. no	0.96 (0.59–1.57)	0.871		
Alcohol history	Yes vs. no	2.57 (1.32–5.01)	0.006	2.83 (1.27–6.34)	0.011
Chronic disease	Yes vs. no	1.21 (0.83–1.77)	0.316		
Liver metastasis	Negative vs. positive	1.52 (0.93–2.47)	0.093		
Lung metastasis	Negative vs. positive	0.81 (0.53–1.24)	0.329		
Bone metastasis	Negative vs. positive	1.30 (0.66–2.58)	0.452		
Peritoneal metastasis	Negative vs. positive	0.86 (0.52–1.43)	0.565		
Tumor site	Rectum/rectosigmoid vs. colon	0.66 (0.45–0.97)	0.036	0.73 (0.42–1.32)	0.302
Blood group	AB vs. others	0.93 (0.16–0.97)	0.042	0.65 (0.30–1.37)	0.254
	RH- vs. RH+	0.60 (0.35–1.05)	0.073		
MNA-SF	≤7 vs. >7	2.29 (1.56–3.36)	<0.001	1.93 (1.09–3.45)	0.025

AC, adenocarcinoma; BMI: body mass index; CI: confidence interval; ECOG PS: Eastern Cooperative Oncology Group Performance Status; HR: Hazard ratio; MNA-SF: Mini Nutritional Assessment-Short Form; PFS: progression-free survival. Statistically significant values are denoted in bold.

### Risk factors for overall survival

The median OS of our patients was 22 months. In the univariate analysis, gender, ECOG PS, smoking history, alcohol history, chronic disease, liver metastasis, lung metastasis, bone metastasis, peritoneal metastasis, and ABO blood group were not significantly associated with OS. In the multivariate analysis, MNA-SF ≤7 points (HR=2.96, 95%CI 1.80–4.89, p<0.001) were identified as negative risk factors for OS. Table 3 shows the factors affecting OS.

**Table 3:** Univariate and multivariate analyses of characteristic parameters related to overall survival.

OS		Univariate analysis		Multivariate analysis	
Characteristics	Category	HR (95%CI)	p	HR (95%CI)	p
Age	<65 vs. ≥65	1.56 (1.01–2.42)	0.046	1.54 (0.96–2.47)	0.075
Gender	Female vs. male	1.31 (0.83–2.08)	0.252		
BMI	≤25.3 vs. >25.3	0.58 (0.37–0.90)	0.015	0.72 (0.44–1.17)	0.179
ECOG PS	≤1 vs. >1	1.18 (0.76–1.82)	0.468		
Smoking history	Yes vs. no	1.32 (0.74–2.35)	0.355		
Alcohol history	Yes vs. no	1.59 (0.76–3.32)	0.218		
Chronic disease	Yes vs. no	1.22 (0.79–1.89)	0.38		
Liver metastasis	Negative vs. positive	1.36 (0.78–2.35)	0.276		
Lung metastasis	Negative vs. positive	1.04 (0.65–1.67)	0.864		
Bone metastasis	Negative vs. positive	1.62 (0.78–3.38)	0.194		
Peritoneal metastasis	Negative vs. positive	0.87 (0.48–1.59)	0.656		
Tumor site	Rectum/rectosigmoid vs. colon	0.63 (0.40–0.98)	0.042	0.81 (0.50–1.30)	0.381
Blood group	AB vs. others	1.22 (0.58–2.54)	0.6		
	RH- vs. RH+	0.51 (0.29–0.91)	0.021	0.58 (0.33–1.03)	0.065
MNA-SF	≤7 vs. >7	3.35 (2.11–5.34)	<0.001	2.96 (1.80–4.89)	<0.001

AC: adenocarcinoma; BMI: body mass index; CI: confidence interval; ECOG PS: Eastern Cooperative Oncology Group Performance Status; HR: hazard ratio; MNA-SF: Mini Nutritional Assessment-Short Form; OS: overall survival; SCC: squamous cell carcinoma. Statistically significant values are denoted in bold.

### Kaplan-Meier survival analysis

Patients who were not malnourished at the time of diagnosis had longer median PFS than patients who were malnourished (17 months vs. 8 months; p<0.001). Similarly, patients who were not malnourished at the time of diagnosis had longer median OS than patients who were malnourished (34 months vs. 18 months; p<0.001).

### Best objective response rate

Examining the best response rates during first-line treatment, patients with progressive disease (39 vs. 70%) were more likely to be malnourished than patients with complete (6 vs. 3%), partial (49 vs. 27%), or stable (6 vs. 0%) responses.

## DISCUSSION

This study investigated the effects of clinicopathological characteristics and nutritional status on survival in patients with mCRC receiving first-line chemotherapy. The history of alcohol use and the presence of malnutrition (MNA-SF ≤7) were associated with worse survival outcomes.

Alcohol consumption has consistently been associated with worse outcomes in CRC, although the underlying mechanisms remain unclear. This was supported by the meta-analysis of Cai et al., which found an increased risk of CRC mortality in heavy drinkers^8^, as well as by Walter et al., who reported that alcohol use was associated with shorter survival in CRC patients^9^. Additionally, a systematic review by Sapkota et al. identified alcohol intake as a contributing factor to liver metastasis, which may partially explain the worse prognosis observed in our cohort^10^. In our analysis, it was found that alcohol consumption significantly affected PFS (p=0.011), while its impact on OS was not statistically significant (p=0.218). These findings underscore alcohol as a modifiable risk factor in mCRC.

Malnutrition is a significant concern in mCRC, affecting both treatment outcomes and survival rates. In line with previous studies, our findings show that malnutrition is an independent risk factor for both PFS and OS in mCRC patients. This aligns with the results of several studies, highlighting the detrimental impact of malnutrition on treatment tolerability and survival. For instance, the study by Gallois et al. demonstrated that malnutrition, as assessed by the patient-generated subjective global assessment score, was strongly associated with OS^11^. Similarly, other studies have emphasized the link between malnutrition and worse survival outcomes, as in the study by Barret et al., which reported that severe malnutrition in mCRC patients was associated with reduced OS^12^. Furthermore, a multicenter study by Karabulut et al. found that moderate-to-severe malnutrition was associated with shorter OS^13^. Our findings are consistent with these studies, where malnutrition appears to worsen the efficacy of treatment, highlighting the importance of early nutritional screening and intervention.

The limitations of our study are the small sample size and its prospective design. The major strength of this study is that it is the first attempt to evaluate the prognostic value of MNA-SF in patients with mCRC receiving first-line treatment.

## CONCLUSION

In our study, a history of alcohol use and the presence of malnutrition (MNA-SF ≤7) were associated with poor survival outcomes in patients with mCRC receiving first-line chemotherapy. Specifically, alcohol consumption significantly affected PFS, while malnutrition was identified as an independent risk factor for both PFS and OS. These findings highlight alcohol consumption and malnutrition as modifiable risk factors for mCRC treatment outcomes. Given the importance of early nutritional screening and intervention, future studies are needed to further investigate these factors, particularly the role of malnutrition, in larger, prospective, and randomized controlled trials.

## Data Availability

The datasets generated and/or analyzed during the current study are available from the corresponding author upon reasonable request.
