# ERRATUM: Nutritional status and prognosis in metastatic colorectal cancer: a cohort study

**DOI:** 10.1590/1806-9282.20250238-ER

**Published:** 2026-04-10

**Authors:** José Maria Soares

In the manuscript: Kapagan T, Bulut N, Arslansoy B, Ozcalimli Z, Turkmencalikoglu M, Yontan E, et al.. Nutritional status and prognosis in metastatic colorectal cancer: a cohort study. Rev Assoc Med Bras. 2025;71(6):e20250238. https://doi.org/10.1590/1806-9282.20250238 on page 2:

**Where it reads:**

The endpoint for PFS was defined as clinical or radiological disease progression after starting nivolumab, and the endpoint for OS was defined as death after starting nivolumab or the date of last follow-up.

**It should read:**

The endpoint for PFS was defined as clinical or radiological disease progression after starting 5-FU–based chemotherapy, and the endpoint for OS was defined as death after starting 5-FU–based chemotherapy or the date of last follow-up.

